# Recombinant protein production data after expression in the bacterium *Escherichia coli*

**DOI:** 10.1016/j.dib.2016.02.074

**Published:** 2016-03-04

**Authors:** J. Enrique Cantu-Bustos, Kevin D. Cano del Villar, Teresa Vargas-Cortez, Jose Ruben Morones-Ramirez, Isaias Balderas-Renteria, Xristo Zarate

**Affiliations:** Universidad Autonoma de Nuevo Leon, Facultad de Ciencias Quimicas, Av. Universidad s/n, Ciudad Universitaria, San Nicolas de los Garza, Nuevo Leon 66451, Mexico

**Keywords:** Fusion protein, Affinity tag, *Escherichia coli*, CusF, GST

## Abstract

Fusion proteins have become essential for the expression and purification of recombinant proteins in *Escherichia coli*. The metal-binding protein CusF has shown several features that make it an attractive fusion protein and affinity tag: "Expression and purification of recombinant proteins in *Escherichia coli* tagged with the metal-binding protein CusF" (Cantu-Bustos et al., 2016 [1]). Here we present accompanying data from protein expression experiments; we tested different protein tags, temperatures, expression times, cellular compartments, and concentrations of inducer in order to obtain soluble protein and low formation of inclusion bodies. Additionally, we present data from the purification of the green fluorescent protein (GFP) tagged with CusF, using Ag(I) metal affinity chromatography.

**Specifications table**

**Table:** 

Subject area	*Molecular Biology*
More specific subject area	*Protein expression and purification*
Type of data	*Pictures. Images of gel electrophoresis.*
How data was acquired	*Sodium dodecyl sulfate polyacrylamide gel electrophoresis (SDS-PAGE)*
Data format	*Raw*
Experimental factors	*Fusion proteins: GST, CusF, and SmbP. Inducer concentration, time and temperature during protein expression. Metal affinity chromatography with silver ions Ag(I). Periplasmic or cytoplasmic protein expression.*
Experimental features	*Production levels of soluble protein and inclusion bodies, protein purity.*
Data source location	*San Nicolas de los Garza, Nuevo Leon, Mexico*
Data accessibility	*Data is within this article*

## Value of the data

• The data shows that the production of soluble recombinant proteins in the bacterium *Escherichia coli* can be improved by varying the fusion protein, time, temperature, and concentration of inducer during protein expression.
• The data indicates that recombinant proteins, exemplified here with the red fluorescent protein (RFP), can be expressed in the cellular periplasm when tagged with full-length CusF and SmbP.
• Protein purification data shows that CusF-tagged proteins may be purified using immobilized metal affinity chromatography with Ag(I) ions instead of the most common Cu(II) or Ni(II).

## 1 Data

**Fig. 1:**
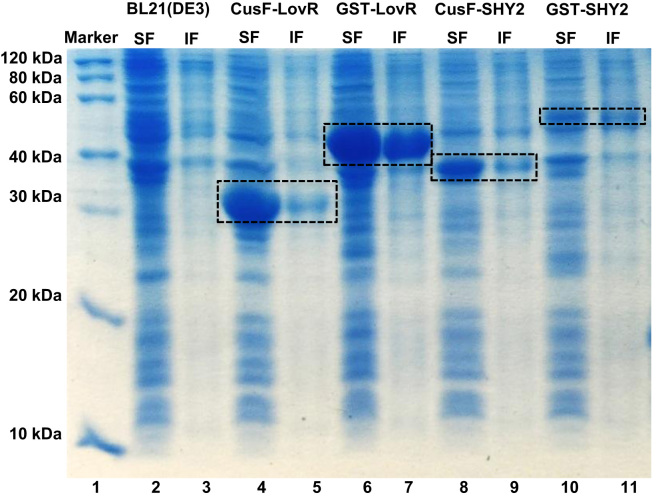
15% SDS-PAGE analysis of small-scale expression for the LovR and SHY2 constructs tagged with CusF and GST at 37 °C, 1 mM IPTG, 4 h. Lane 1: protein marker; lanes 2 and 3: soluble and insoluble fractions of *E. coli* BL21(DE3) lysate control; lanes 4 and 5: soluble and insoluble fractions of CusF-LovR; lanes 6 and 7: soluble and insoluble fractions of GST-LovR; lanes 8 and 9: soluble and insoluble fractions of CusF-SHY2; lanes 10 and 11: soluble and insoluble fractions of GST-SHY2. Calculated molecular weights for CusF-LovR: 27.4 kDa, GST-LovR: 43.6 kDa, CusF-SHY2: 33.1 kDa, and GST-SHY2: 49.1 kDa.

**Fig. 2:**
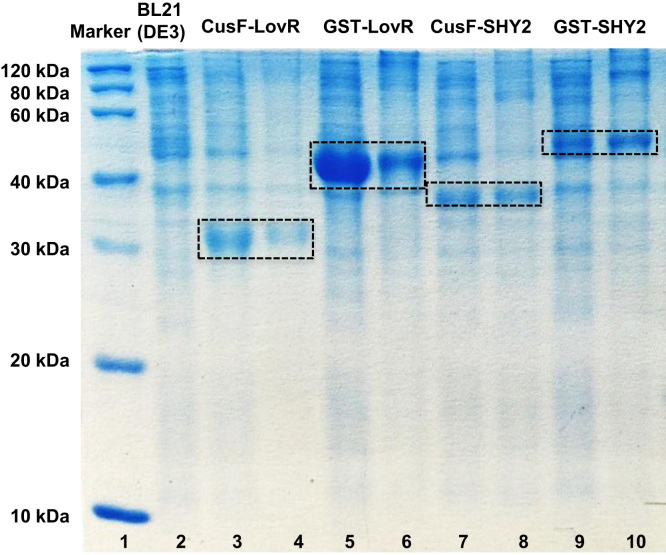
15% SDS-PAGE analysis of the insoluble fractions (inclusion bodies) produced from small-scale expressions at 37 °C and 25 °C for the LovR and SHY2 constructs tagged with CusF and GST. Lane 1: molecular weight marker; lane 2 *E. coli* BL21(DE3) background control; lanes 3 and 4: insoluble fractions of CusF-LovR at 37 °C and 25 °C respectively; lanes 5 and 6: insoluble fractions of GST-LovR at 37 °C and 25 °C respectively; lanes 7 and 8: insoluble fractions of CusFc-SHY2 at 37 °C and 25 °C respectively; lanes 9 and 10: insoluble fractions of GST-SHY2 at 37 °C and 25 °C respectively.

Fig. 1 shows the SDS-PAGE analysis comparing soluble and insoluble protein content for the proteins LovR and SHY2 tagged with two different protein tags and expressed at different conditions. Additionally, the electrophoretic analysis in Fig. 2 compares the content of inclusion bodies.

**Fig. 3:**
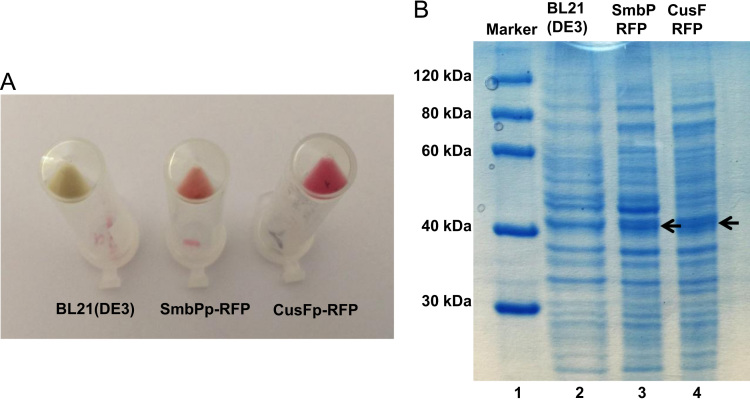
Expression of red fluorescent protein in the periplasm of *E. coli* tagged with SmbPp and CusFp. (A) Picture of *E. coli* BL21(DE3) cell pellets after expression of the different RFP constructs. The protein tag used for each construct is indicated below. A control, uninduced *E. coli* cells on the left, is included to show regular cell color. (B) 10% SDS-PAGE analysis of RFP periplasmic expression. Lane 1: protein marker; lane 2: *E. coli* BL21(DE3) periplasmic background control; lane 3: SmbPp-RFP periplasmic fraction; lane 4: CusFp-RFP periplasmic fraction. Arrows indicate the band for SmbPp-RFP and CusFp-RFP. Calculated molecular weights for SmbPp-RFP: 37.2 kDa, CusFp-RFP: 38.1 kDa.

Recombinant proteins tagged with the protein tags CusFp and SmbPp, containing their signal sequences, are exported to the cell׳s periplasm. Fig. 3 shows an image of *E. coli* BL21(DE3) cells after RFP expression and the electrophoretic analysis of the periplasmic lysates.

**Fig. 4:**
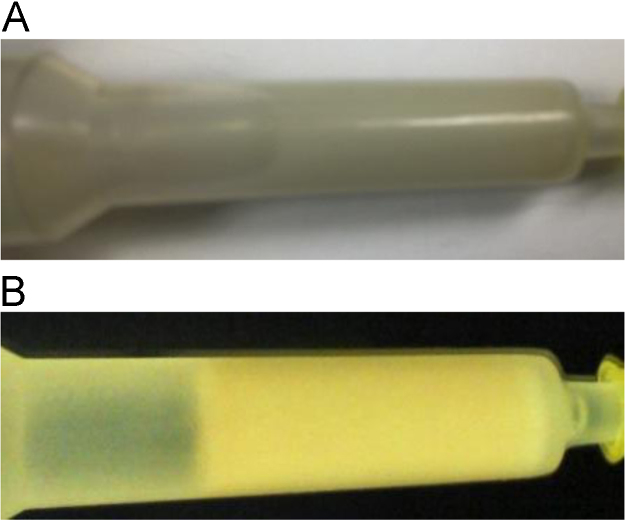
Binding of CusF-GFP to Ag(I) chromatography media. (A) Image of the functionalized Bio-Gel P2 polyacrylamide media with glutaraldehyde and thiourea for immobilization of Ag(I) ions. (B) the same Ag(I) media after incubation with the lysate from *E. coli* expressing the CusF-GFP construct.

**Fig. 5:**
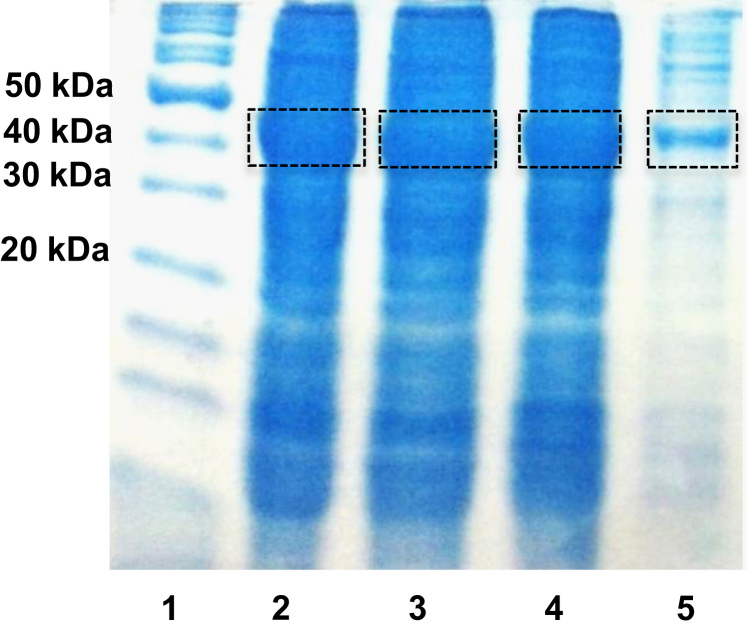
14% SDS-PAGE analysis of the purification of CusF-GFP with Ag(I) affinity chromatography. Lane 1: protein marker; lane 2: lysate; lane 3: flow-through; lane 4: first elution fraction with 160 mM methionine; lane 5: second elution fraction. Calculated molecular weight for CusF-GFP: 40.8 kDa.

Fig. 4 shows pictures of the synthesized silver chromatographic media before and after incubation with the *E. coli* lysate expressing green fluorescent protein tagged with CusF. Fig. 5 shows the SDS-PAGE analysis of the purification steps, it shows the protein content in the flow-through (the lysate after incubation with the Ag(I) resin), and two elutions steps with 160 mM methionine.

## 2 Experimental design, materials and methods

### 2.1 DNA constructs

Full-length CusF (CusFp, for periplasmic expression) was amplified with primers 5′-AGTCAGTCA**CATATG**AAAAAAGCACTGCAAGTCG-3′ (NdeI, forward) and 5′-ATGCATGCA**GGTACC**CTGGCTGACTTTAATATCCTGTAA-3′ (KpnI, reverse). The 50 μL reaction comprised 10 ng of template DNA, 60 pmol of each primer, 1.5 μL of 10 mM dNTPs mix, and 2 units of Vent DNA polymerase (New England Biolabs) in 1× ThermoPol reaction buffer. The thermocycler conditions were 95 °C for 2 min; 30 cycles of 95 °C–1 min, 59 °C–1 min, 72 °C–1 min; and a final extension at 72 °C for 10 min. Amplification of CusF lacking the signal sequence (for cytoplasmic expression) was done with forward primer 5′-AGTCAGTCA**CATATG**GCTAACGAACATCATCATGAAAC-3′ (NdeI) and the same reverse primer and thermocycler conditions as before. pET30a vector was linearized with NdeI and KpnI, and the CusF insert was ligated into it following digestion with the same enzymes. The gene for GST was cloned with the same restriction sites for comparative studies. Target proteins were cloned using NcoI and XhoI restriction sites, in order to place the enterokinase recognition sequence between CusF and the target protein for tag removal. The target proteins expressed in the cellular cytoplasm (using the CusF construct) were LovR from *Caulobacter crescentus* [2], the *Arabidopsis thaliana* protein Short Hypocotyl 2 (SHY2) [3], and the S65T mutant of Green Fluorescent Protein (GFP) [4]. Red Fluorescent Protein [5], was expressed in the periplasm with the CusFp construct. A second RFP construct was tested for expression in the periplasm but tagged with SmbPp as previously described [6].

### 2.2 Protein expression

Recombinant proteins produced in *E. coli* are typically expressed at 37 °C for 3–4 h and induced with 1.0 mM isopropyl β-D-1-thiogalactopyranoside (IPTG) to deactivate the LacI repressor; these conditions sometimes form insoluble inclusion bodies depending on the target protein [7]. The proteins LovR and SHY2, tagged with CusF and GST at the N-terminal, were expressed at the conditions mentioned above and alternatively at 25 °C, 16 h with 0.1 mM IPTG to avoid aggregation. DNA constructs were transformed into *E. coli* BL21(DE3). 2 mL of Luria–Bertani broth (with 30 μg/mL kanamycin) was inoculated with a single colony and incubated at 37 °C and 220 rpm until an OD_600_ of 0.5 was attained. IPTG was added to a final concentration of 0.1 mM to induce expression; cells were incubated overnight at room temperature (25 °C) and 220 rpm. If expression was induced with 1.0 mM IPTG, the cells were incubated at 37 °C for 4 h and 220 rpm. After protein expression cells were harvested by centrifugation and resuspended in 100 μL 1× SDS-PAGE sample buffer, incubated in boiling water for 10 min, and then centrifuged for 10 min at 13,000 rpm; the supernatant was used for soluble protein content analysis with SDS-PAGE. The residual pellet was resuspended in 100 μL 8 M urea buffer and boiled for 10 min to solubilize the inclusion bodies, then centrifuged at the same speed; the supernatant was used for the analysis of insoluble protein content with SDS-PAGE. For CusF-GFP large-scale expression, cells were grown in baffled flasks until OD_600_ reached 0.5, expression was induced by adding IPTG up to 0.1 mM, cells were incubated overnight at room temperature (25 °C) and 220 rpm. For large-scale expression of CusFp-RFP and SmbPp-RFP expression, cells were grown in the same manner.

### 2.3 *E. coli* periplasm lysis

Expression of recombinant proteins in the cellular periplasm has certain advantages since it contains less proteases [8], and provides the proper conditions for the formation of disulfide bonds [9]. If the protein of interest is tagged with the CusFp or SmbPp constructs that include the signal sequences, then the target protein is exported to the periplasm. An osmotic shock procedure was performed to obtain the periplasmic fraction. Cells from a 1 L culture were resuspended in 20 mM Tris–HCl, 30% sucrose, 2.5 mM EDTA, pH 8.0 (5 mL/g of cell pellet) and incubated on ice for 1 h. Cells were centrifuged at 10,000 rpm for 15 min at 4 °C, the supernatant was recovered and saved, and the sediment was resuspended in 20 mL ice-cold 5 mM MgSO_4_ buffer for 1 h. Cells were centrifuged at 10,000 rpm for 15 min at 4 °C, and the supernatant was also considered as periplasmic fraction, the two supernatants were pooled, and analyzed by SDS-PAGE.

### 2.4 Purification of CusF-GFP with immobilized Ag(I) affinity chromatography

CusF is a metal-binding protein with high binding affinities to copper and silver ions, especially Cu(I) and Ag(I) [10]; although it has been demonstrated that binds Cu(II) as well [11]. This last feature has allowed the purification of CusF-tagged proteins with immobilized Cu(II) affinity chromatography [1]. Nevertheless, the first metal ion tested to purify CusF-tagged proteins was Ag(I); this metal ion was immobilized on a functionalized acrylamide resin with glutaraldehyde and thiourea. Polyacrylamide beads (Bio-Gel P2, from BIO-RAD) were functionalized with silver ions as previously described [12]. First, a 25% glutaraldehyde solution was polymerized for 24 h at 50 °C. After hydration of the Bio-Gel P2 beads with a 50 mM sodium phosphate buffer (pH 8.0) for 24 h, the polymerized glutaraldehyde solution was added to the wet resin (gel) in a ratio of 20 mL/1 g of dry gel. They were incubated at 37 °C/120 rpm for 24 h, afterwards the resin was filtered and rinsed with deionized water. For every 1 g of initial dry gel, 20 mL of 1.0 M thiourea solution was added to the glutaraldehyde-activated gel resin and the slurry was incubated at 37 °C/120 rpm. After 24 h of reaction, the thiourea solution was removed by decantation and the gel was filtered and washed with deionized water again. The final step involved the addition of silver nitrate in a ratio of 0.9 mL of 1.0 N AgNO_3_ for 1 g of dry gel and the solution was incubated at 37 °C. After 24 h the resin was washed with deionized water to remove all the unbounded silver. Cells expressing CusF-GFP were harvested by centrifugation at 4 °C and then resuspended in ice-cold lysis buffer (50 mM sodium phosphate, 300 mM NaCl, pH 8.0). Cells were lysed using a bead-beater and 0.1 mm glass beads (both from BioSpec Products); the lysate was clarified by centrifugation at 4 °C, 15,000 rpm for 20 min. 1 mL of the Ag(I) resin was incubated with the 5 mL of CusF-GFP lysate 30 min at room temperature. The resin was washed thoroughly with lysis buffer. Since two methionines and one histidine are used by CusF to bind Ag(I) [13], elution steps were performed using a buffer with a high methionine concentration (50 mM sodium phosphate, 300 mM NaCl, 160 mM methionine, pH 8.0). Elution fractions were analyzed by SDS-PAGE.

## References

[1] Cantu-Bustos J.E., Vargas-Cortez T., Morones-Ramirez J.R., Balderas-Renteria I., Galbraith D.W., McEvoy M.M. (2016). Expression and purification of recombinant proteins in *Escherichia coli* tagged with the metal-binding protein CusF. Protein Expr. Purif..

[2] Foreman R., Fiebig A., Crosson S. (2012). The *lovK-lovR* two-component system is a regulator of the general stress pathway in *Caulobacter crescentus*. J. Bacteriol..

[3] Kim B.C., Soh M.S., Hong S.H., Furuya M., Nam H.G. (1998). Photomorphogenic development of the *Arabidopsis* shy2-1D mutation and its interaction with phytochromes in darkness. Plant J..

[4] Heim R., Tsien R.Y. (1996). Engineering green fluorescent protein for improved brightness, longer wavelengths and fluorescence resonance energy transfer. Curr. Biol..

[5] Campbell R.E., Tour O., Palmer A.E., Steinbach P.A., Baird G.S., Zacharias D.A. (2002). A monomeric red fluorescent protein. Proc. Natl. Acad. Sci. U.S.A..

[6] Vargas-Cortez T., Morones-Ramirez J.R., Balderas-Renteria I., Zarate X. (2016). Expression and purification of recombinant proteins in *Escherichia coli* tagged with a small metal-binding protein from *Nitrosomonas europaea*. Protein Expr. Purif..

[7] Rosano G.L., Ceccarelli E.A. (2014). Recombinant protein expression in *Escherichia coli:* advances and challenges.. Front. Microbiol..

[8] Dow B.A., Tatulian S.A., Davidson V.L. (2015). Use of the amicyanin signal sequence for efficient periplasmic expression in *E. coli* of a human antibody light chain variable domain. Protein Expr. Purif..

[9] Rastgar Jazii F., Karkhane A.A., Yakhchali B., Fatemi S.S., Deezagi A. (2007). A simplified purification procedure for recombinant human granulocyte macrophage-colony stimulating factor from periplasmic space of *Escherichia coli*. J. Chromatogr. B Anal. Technol. Biomed. Life Sci..

[10] Kittleson J.T., Loftin I.R., Hausrath A.C., Engelhardt K.P., Rensing C., McEvoy M.M. (2006). Periplasmic metal-resistance protein CusF exhibits high affinity and specificity for both CuI and AgI. Biochemistry.

[11] Astashkin A.V., Raitsimring A.M., Walker F.A., Rensing C., McEvoy M.M. (2005). Characterization of the copper(II) binding site in the pink copper binding protein CusF by electron paramagnetic resonance spectroscopy. J. Biol. Inorg. Chem..

[12] Garcia A.A., Kim D.H., Miles D.R. (1994). Immobilization of silver and platinum ions for metal affinity chromatography. React. Polym..

[13] Loftin I.R., Franke S., Blackburn N.J., McEvoy M.M. (2007). Unusual Cu(I)/Ag(I) coordination of *Escherichia coli* CusF as revealed by atomic resolution crystallography and X-ray absorption spectroscopy. Protein Sci..

