# Supplementary material for: Recombinant protein production data after expression in the bacterium Escherichia coli
Source: Data Brief. 2016 Mar 4;7:502–8. doi: 10.1016/j.dib.2016.02.074 (PMC4792856; doi:10.1016/j.dib.2016.02.074)
Supplement: Supplementary file 1 — Supplementary material [file mmc1.doc]

**Dr. Xristo Zarate Kalfopulos**

**Facultad de Ciencias Quimicas**

**Universidad Autonoma de Nuevo Leon**

**Av. Universidad s/n**

**Cd. Universitaria**

**San Nicolas de los Garza, NL 66451**

**Mexico**

February 12, 2016

**Editor-in-Chief**

**Data in Brief**

Dear editor,

Please find attached the revised manuscript entitled *“Recombinant protein production data after expression in the bacterium Escherichia coli”* to be considered for publication in *Data in Brief.*

We have addressed all the reviewer's comments and recommendations. Furthermore, we want to state that **there are no conflicts of interest**.

Sincerely yours,

Xristo Zarate Kalfopulos

xristo.zaratekl@uanl.edu.mx

+52 81 83294000 ext. 3444
